# The importance of retaining physical functions to prevent skeletal‐related events in multiple myeloma patients with bone disease

**DOI:** 10.1002/jha2.402

**Published:** 2022-02-28

**Authors:** Hirokazu Miki, Shingen Nakamura, Masahiro Oura, Masafumi Nakamura, Ryohei Sumitani, Kimiko Sogabe, Mamiko Takahashi, Tomoko Maruhashi, Takeshi Harada, Shiro Fujii, Hirofumi Hamano, Masateru Kondo, Naoto Okada, Itsuro Endo, Masahiro Abe

**Affiliations:** ^1^ Division of Transfusion Medicine and Cell Therapy Tokushima University Hospital Tokushima Japan; ^2^ Department of Community Medicine and Medical Science Tokushima University Graduate School of Biomedical Sciences Tokushima Japan; ^3^ Department of Hematology Endocrinology and Metabolism, Institute of Biomedical Sciences Tokushima University Graduate School Tokushima Japan; ^4^ Clinical Research Center for Developmental Therapeutics Tokushima University Hospital Tokushima Japan; ^5^ Department of Pharmacy Tokushima University Hospital Tokushima Japan

**Keywords:** amyloidosis, bone disease, denosumab, myeloma, skeletal‐related events

## Abstract

This study was undertaken to identify baseline conditions and triggering factors for skeletal‐related events (SRE) in multiple myeloma (MM) patients treated with denosumab. During the median follow‐up of 17 months, SRE occurred in 6 out of 52 newly diagnosed patients and in 5 out of 23 relapsed/refractory patients. Bone fractures occurred by falling down due to orthostatic hypotension and/or muscle weakness in three out of four cases with amyloid light‐chain (AL) amyloidosis. A loss of balance and falling down appear to be triggering factors for SRE, especially in frail MM patients with AL amyloidosis, indicating the importance of retaining physical functions to prevent SRE.

## 1 INTRODUCTION

Multiple myeloma (MM) develops and expands almost exclusively in the bone marrow and generates devastating bone destruction. Zoledronic acid is widely used for the prevention of skeletal‐related events (SRE) in MM [1, 2]. A large phase 3 study demonstrated the noninferiority of denosumab to zoledronic acid in newly diagnosed MM (NDMM) patients with at least one bone lesion in terms of the prevention of SRE [3]. Overall survival was similar between the denosumab and zoledronic acid arms; of note, progression‐free survival (PFS) was longer with denosumab than zoledronic acid [3]. According to the subgroup analysis of this study, most of MM patients were treated with proteasome inhibitor (PI)‐based regimens, and PFS benefits with denosumab were observed in NDMM who underwent autologous stem cell transplantation and those who received PI‐based regimens, suggesting that denosumab may prolong anti‐MM efficacy in combination with PI‐based induction therapy [4]. PI‐based regimens combined with denosumab might provide a potentially synergistic anti‐MM effects and prevent the occurrence of SRE. Although MM tumor progression is generally accepted to be among the major causative factors for SRE in MM patients, baseline conditions and triggering factors for SRE in MM patients remain largely unknown. The present study was undertaken to identify triggering factors or the underlying physical functions associated with SRE in MM patients treated with denosumab.

### 1.1 Patients and methods

We retrospectively analyzed 75 MM patients who received a subcutaneous injection of 120 mg denosumab for bone disease between June 2012 and December 2020 in Tokushima University Hospital. The present study was approved by the Institutional Review Board of Tokushima University (permission number 3086‐2). The bone scale was determined as previously reported [5]. The documentation of a lytic bone lesion was based on radiographic (X‐ray or CT) evidence of at least one lytic bone lesion. SRE were defined as one or more of the following: a pathologic fracture (vertebral or nonvertebral), radiation therapy to bone, surgery to bone, or spinal cord compression. We assessed the time to the first SRE after the denosumab treatment using medical charts and showed Kaplan–Meier estimates of the time to the first SRE in our cohort. Univariate and multivariate analyses of risk factors for SRE were performed using Fisher's exact test and a logistic regression analysis. The proportion of MM patients without SRE was estimated using the Kaplan–Meier method and analyzed using the Log‐rank test. Data were analyzed using JMP13.0 (SAS Institute Inc.). *p* values < 0.05 were considered statistically significant.

## 2 RESULTS AND DISCUSSION

**FIGURE 1:**
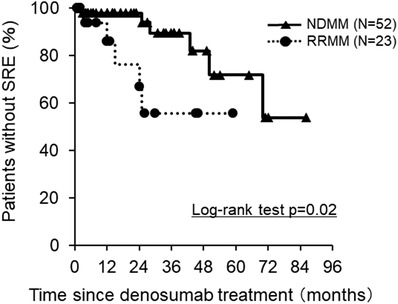
Kaplan–Meier estimates of the time to the first SRE on this study. The proportion of patients without SRE according to the disease status at the first administration of denosumab, NDMM (N = 52, solid line) and RRMM (N = 23, dotted line)

Patient characteristics at the first administration of denosumab are shown in Table 1. Fifty‐two patients with NDMM and 23 with relapsed/refractory MM (RRMM) were enrolled. The severity of bone disease at baseline was as follows: 13, 45, and 17 patients were scored as bone scale 1, 2, and 3, respectively, according to the Durie and Salmon criteria [5]. Fifteen were in poor performance status (PS) with Eastern Cooperative Oncology Group (ECOG) 3 and more, and 4 were combined with amyloid light‐chain (AL) amyloidosis. All patients were treated with PI‐based regimens at least one line in their clinical courses. The number of times denosumab was administered ranged between 1 and 35 (median 7). At the median follow‐up of 17 months (interquartile range [IQR]: 1–86), SRE occurred in 6 out of 52 patients with NDMM and 5 out of 23 with RRMM. Patients with RRMM had a significantly shorter time to the first occurrence of SRE than those with NDMM (Figure 1). The proportion of patients without SRE at 3 years was 89.6% in NDMM and 55.7% in RRMM (Log‐rank test, *p* = 0.02) (Figure 1). We next exploited the baseline physical conditions and triggering factors for SRE. A univariate analysis revealed that PS with ECOG 3 and more (odds ratio: 4.50, 95% CI: 1.15–17.63, *p* = 0.037) and the coexistence of AL amyloidosis (odds ratio: 23.63, 95% CI: 2.19–255.21, *p* = 0.009) correlated with the occurrence of SRE (Table 2, upper panel). In the multivariate analysis, the coexistence of AL amyloidosis remained an independent risk factor for SRE occurrence (odds ratio: 14.62, 95% CI:1.20–177.50, *p* = 0.035) (Table 2, lower panel).

**TABLE 1:** Patient characteristics

Sex (male/female)	38/37
Median age (range), years	69 (44‐88)
Newly diagnosed	52 (69%)
Relapsed/refractory	23 (31%)
Immunoglobulin subtype	
IgG	46 (61%)
IgA	12 (16%)
IgD	3 (4%)
Light chain only	13 (17%)
Nonsecretory	1 (1%)
PS (ECOG)	
0	26 (35%)
1	24 (32%)
2	10 (13%)
3	10 (13%)
4	5 (7%)
Durie and Salmon stage	
Ⅰ	0 (0%)
Ⅱ	23 (31%)
III	52 (69%)
A	61 (81%)
B	14 (19%)
ISS stage	
1	24 (32%)
2	28 (37%)
3	23 (31%)
Bone scale	
1	13 (17%)
2	45 (60%)
3	17 (23%)
Previous bisphosphonate treatment	16 (21%)
History of DM	17 (23%)
History of SRE	34 (45%)
AL amyloidosis	4 (5%)
Anti‐myeloma treatment	
Proteasome inhibitors	75 (100%)
IMiDs	51 (68%)
ASCT	25 (33%)

ASCT, autologous stem cell transplantation; DM, diabetes mellitus; ECOG, Eastern Cooperative Oncology Group; IMiDs, immunomodulatory drug; ISS: international staging system; PS, performance status; SRE, skeletal‐related events,

**TABLE 2:** Risk factors for SRE

Univariate analysis					
Variables		SRE−	SRE+	95% C.I./Odds raio	*p*‐value
Age	<65 ≥65	19 45	3 8	0.27‐4.71 1.13	1.0000
Sex	male female	31 33	7 4	0.14‐2.01 0.54	0.5161
Previous BP treatment	no yes	51 13	8 3	0.34‐6.33 1.47	0.6922
Bone scale	0, 1 2, 3	12 52	1 10	0.93‐17.30 4.00	0.1009
History of DM	no yes	52 12	6 5	0.94‐13.83 3.61	0.1110
History of SRE	no yes	35 29	6 5	1.25‐24.43 1.01	1.0000
PS (ECOG)	0, 1, 2 3, 4	54 10	6 5	1.15‐17.63 4.50	0.0370
AL amyloidosis	no yes	63 1	8 3	2.19‐255.21 23.63	0.0090

Multivariate analysis				
Variables		95% C.I.	Odds ratio	*p*‐value
PS (ECOG)	3, 4	0.58 – 12.90	2.74	0.2030
AL amyloidosis	yes	1.20 – 177.50	14.62	0.0352

BP, bisphosphonate; CI, confidence interval; DM, diabetes mellitus; ECOG, Eastern Cooperative Oncology Group; PS, performance status.

SRE occurred in 11 MM patients in our cohort. Among 8 patients without documented AL amyloidosis, 7 showed progressive disease and 1 stable disease (Table 3, patients # 1–8). Falling down and a loss of balance caused bone fractures in 3 patients whose PS were ECOG 2 and more (Table 3, patients # 1, 3, and 7). Importantly, 3 MM patients with AL amyloidosis developed SRE triggered by falling down and/or a loss of balance, although they achieved very good partial response (Table 3, patient # 9–11). Two of these patients suffered from cardiac amyloidosis with orthostatic hypotension and muscle weakness or peripheral neuropathy (Table 3, patients # 9 and 11).

**TABLE 3:** Characteristics of MM patients when SRE occurred

No.	Age/Sex	Amyloidosis	Details of SRE	Treatment response	Triggers	Complications/Comorbidities	PS (ECOG)
1	76/M	None	Femoral fracture	SD	Loss of balance Falling down	Peripheral neuropathy	2
2	47/M	None	Rib fracture Spinal cord compression	PD	None	None	2
3	56/M	None	Pelvic fracture	PD	Loss of balance Falling down	DM type 2 Orthostatic hypotension	4
4	55/M	None	Vertebral fracture Spinal cord compression	PD	None	Peripheral neuropathy	4
5	78/M	None	Sacral fracture Spinal cord compression	PD	None	None	3
6	77/M	None	Vertebral fracture Rib fracture	PD	None	DM type 2 Orthostatic hypotension	1
7	74/F	None	Vertebral fracture	PD	Loss of balance	Peripheral neuropathy	3
8	80/F	None	Vertebral fracture	PD	None	DM type 2 Muscle weakness	3
9	73/F	Heart, Tongue, Skin	Femoral fracture	VGPR	Falling down	Muscle weakness Orthostatic hypotension	2
10	78/F	GI tract, Skin, Muscle	Vertebral fracture	VGPR	Loss of balance Falling down	Muscle weakness	1
11	70/M	Heart, Kidney, GI tract	Rib fracture	VGPR	Falling down	DM type 2 Peripheral neuropathy Orthostatic hypotension	0

DM, diabetes mellitus; ECOG, Eastern Cooperative Oncology Group; F, female; GI tract, gastrointestinal tract; M, male; PD, progressive disease; PS, performance status; SD, stable disease, VGPR, very good partial response.

The occurrence of new SRE was documented in more than 60% of NDMM patients within the first 3 months in a pivotal phase 3 study [3]. Achieving quick and deep response is needed to prevent the early occurrence of SRE and the progression of bone disease in MM. However, a loss of balance and falling down due to ill physical functioning, including muscle weakness, sarcopenia, orthostatic hypotension, and peripheral neuropathy, appear to be triggering factors for SRE, indicating the importance of retaining physical functions in MM patients with bone disease. We need to pay much attention to avoid falling down and a loss of balance especially in frail MM patients and those with AL amyloidosis, although avoiding falling down is a trite and obvious remark.

However, there are some limitations in the present study that include (1) a small sample size especially with the very small number of cases with AL amyloidosis, (2) a retrospective analysis in a single center, and (3) no objective estimation of physical functions. A large multicenter prospective study should be conducted to further elucidate the importance of physical functions to prevent the occurrence of SRE especially in frail MM patients including those with AL amyloidosis.

## AUTHOR CONTRIBUTIONS

H.M., S.N., I.E., and M.A. designed the study and wrote the manuscript. All authors were involved in the analyses and interpretation of data. All authors approved the submission of the manuscript.

## CONFLICT OF INTEREST

M.A. received honoraria for lectures from Daiichi Sankyo Company Limited. Other authors declare no competing financial interests related to this work.
